# PACAP Neurons in the Ventromedial Hypothalamic Nucleus Are Glucose Inhibited and Their Selective Activation Induces Hyperglycaemia

**DOI:** 10.3389/fendo.2018.00632

**Published:** 2018-10-30

**Authors:** Tansi Khodai, Nicolas Nunn, Amy A. Worth, Claire H. Feetham, Mino D. C. Belle, Hugh D. Piggins, Simon M. Luckman

**Affiliations:** ^1^Faculty of Biology, Medicine and Health, University of Manchester, Manchester, United Kingdom; ^2^Medical School, University of Exeter, Exeter, United Kingdom

**Keywords:** PACAP, VMH, glucose sensing, glucose inhibited, hypoglycaemia

## Abstract

**Background:** Glucose-sensing neurons are located in several parts of the brain, but are concentrated in the ventromedial nucleus of the hypothalamus (VMH). The importance of these VMH neurons in glucose homeostasis is well-established, however, little is known about their individual identity. In the present study, we identified a distinct glucose-sensing population in the VMH and explored its place in the glucose-regulatory network.

**Methods:** Using patch-clamp electrophysiology on *Pacap*-cre::EYFP cells, we explored the glucose-sensing ability of the pituitary adenylate cyclase-activating peptide (PACAP) neurons both inside and outside the VMH. We also mapped the efferent projections of these neurons using anterograde and retrograde tracing techniques. Finally, to test the functionality of PACAP^VMH^ *in vivo*, we used DREADD technology and measured systemic responses.

**Results:** We demonstrate that PACAP neurons inside (PACAP^VMH^), but not outside the VMH are intrinsically glucose inhibited (GI). Anatomical tracing techniques show that PACAP^VMH^ neurons project to several areas that can influence autonomic output. *In vivo*, chemogenetic stimulation of these neurons inhibits insulin secretion leading to reduced glucose tolerance, implicating their role in systemic glucose regulation.

**Conclusion:** These findings are important as they identify, for the first time, a specific VMH neuronal population involved in glucose homeostasis. Identifying the different glucose-sensing populations in the VMH will help piece together the different arms of glucose regulation providing vital information regarding central responses to glucose metabolic disorders including hypoglycaemia.

## Introduction

Glucose is the primary source of energy for the brain, so it is not surprising that several redundant neural pathways are involved in the detection and tight regulation of this nutrient. Rapid changes in glucose are detected by specialized glucose-sensing neurons which are distributed through different parts of the brain. However, the densest concentration of glucose-sensing neurons is found in the ventromedial hypothalamus and, specifically in the ventromedial nucleus (VMH), though little is known about their identity. A specific role for the VMH in glucoregulation has been described in classic rat experiments (1–3) and confirmed, more recently, by using steroidogenic factor (SF-1) to selectively target this nucleus in the mouse. The deletion of vesicular protein, vGLUT2, in SF-1 neurons produces a marked defect in glucagon secretion in response to fasting or hypoglycaemia (4), while the chemogenetic or electromagnetic activation of SF-1 neurons, as well as optogenetic activation of projections both to and from the VMH cause hyperglycaemia (5–7).

The VMH is a complex structure containing many different cell phenotypes, and work in the rat has identified a range of neuronal responses to the application of glucose (8). Approximately, 14% of rat VMH neurons are inherently sensitive to increased glucose and respond with an increase in electrical activity. These are termed glucose excited (GE) and are thought to depend on glucose metabolism and inhibition of an ATP-sensitive potassium channel for their intrinsic sensing ability (8, 9). A much smaller subset (about 3% of VMH neurons in the rat) reduce their firing in response to glucose and are termed glucose inhibited (GI). Inherent GI neurons in the VMH are dependent on an AMP-activated protein kinase which triggers the production of nitric oxide by the synthetic enzyme (NOS) which may, in turn, reduce a membrane chloride conductance (8–11). Importantly, 14% of VMH neurons are also depolarized by reduced glucose, but this is dependent on synaptic transmission. Thus, these have been termed presynaptically excited by decreased glucose (PED), but it is not clear whether their synaptic drive comes from within or from outside the VMH (or perhaps both). Another 19% of VMH neurons respond to changes in glucose much higher than normally experienced *in vivo*, and so the physiological relevance of these to glucoregulation is debateable (8). Neurons that project information from the VMH may or may not themselves be intrinsically glucose sensing; they may respond in opposing directions to changes in glucose; and, perhaps two thirds of VMH neurons may have no direct role in affecting glucose. Therefore, manipulation of a mixed SF-1 population is likely to produce a complex, “compound” effect.

We have shown previously that a population of VMH neurons co-localize SF-1 and pituitary adenylate cyclase-activating peptide (PACAP), and that the expression of the latter is sensitive to energy status (12). Also, work done by Kalsbeek and colleagues has shown that PACAP from an unknown source, acting through pre-autonomic neurons in the hypothalamic paraventricular nucleus (PVH) can initiate endogenous glucose production (13). In the current paper, we demonstrate that PACAP^VMH^ neurons are GI, and project to different regions of the brain, including the PVH, thought to be involved in the response to hypoglycaemia. Furthermore, selective chemogenetic activation of PACAP^VMH^ neurons can inhibit insulin secretion and increase circulating glucose. This is the first demonstration of a single, defined population of VMH glucose-sensing neuron.

## Materials and methods

### Animals

All experiments were performed in accordance with the UK Animals (Scientific Procedures) Act 1986, and approved by the University of Manchester Animal Welfare and Ethics Review Board. *Pacap*-*IRES*-cre (*Pacap*-cre) mice (kind gift from Prof. Brad Lowell, Harvard Medical School, USA and Dr Michael Krashes, National Institute of Health, USA) were generated and described elsewhere (14). *Pacap*-cre mice were used for *in vivo* physiological studies or crossed with a Rosa26-EYFP reporter line (15) to generate *Pacap*-cre::EYFP mice for electrophysiological recordings. C57BL/6J mice were obtained from Envigo, UK. All mice were housed in groups of 2–5 with *ad libitum* access to chow (Special Diet Services, Witham, UK) and water. They were maintained on a 12 h light/dark cycle, at 22±1°C and 45±10% humidity. Electrophysiological recordings were carried out on slices prepared from 6 to 8 week-old male or female mice, and *in vivo* studies were performed on 8–12 week-old male mice.

### Electrophysiological recordings

#### Brain slice preparation

After sacrificing the animal, brain tissue was extracted and placed in ice-cold, pre-gassed (with 95% O_2_ and 5% CO_2_), artificial cerebrospinal fluid (CSF) containing 95 mM NaCl, 1.8 mM KCl, 1.2 mM KH_2_PO_4_, 7 mM MgSO_4_, 26 mM NaHCO_3_, 15 mM glucose, 50 mM sucrose, 0.5 mM CaCl_2_, and 0.005 mg/L phenol red. 250 μm thick coronal slices were cut using a 7000smz vibrating microtome (Campden Instruments, Loughborough, UK) and maintained in artificial CSF for at least 2 h at room temperature before recording.

#### Whole-cell patch-clamp recording

Prior to recording, the slice containing the VMH was transferred into a recording solution containing 127 mM NaCl, 1.8 mM KCl, 1.2 mM KH_2_PO_4_, 1.3 mM MgSO_4_, 26 mM NaHCO_3_, 2.5 mM glucose, and 0.005 mg/L phenol red. Patch pipettes were filled with 130 mM K-gluconate, 10 mM KCl, 2 mM MgCl_2_, 10 mM HEPES, 0.5 mM EGTA, 2 mM K_2_ATP, 0.5 mM NaGTP (pipette resistance 7 Ω). PACAP cells were visualized using a fluorescent microscope (Olympus BX50WI, Japan). Current-clamp recordings were amplified using AXOCLAMP-2A (Axon Instruments, CA, USA) and acquired via a CED 1401 mk1 A/D interface, controlled by Spike2 software (version 6, Cambridge Electronic Design Limited, Cambridge, UK). Data analysis was carried out using Spike2 software and plotted using Prism (version 7, GraphPad Software, La Jolla California USA). Data are represented as mean ± SEM. Firing frequency is normalized against baseline firing frequency.

#### Applications

Recordings were allowed to stabilize at 2.5 mM glucose for at least 5 min after which 1 mM glucose was applied to the bath. Following application, the solution was once again changed to 2.5 mM during the wash-out period. To measure intrinsic activity, 1 μM tetrodotoxin (TTX) (Cat. No.1069, Tocris Biosciences, Bristol, UK) in 2.5 mM glucose was applied to the bath followed by a 1 μM TTX in 1 mM glucose for 3 to 5 min. 100 nM cholecystokinin (CCK-8S, Tocris Biosciences, Bristol, UK) was also applied in 2.5 mM glucose.

### Surgery

The designer receptors exclusively activated by designer drugs (DREADDs) were delivered by the adeno-associated viruses, AAV8/hSyn-DIO-hM3D(Gq) tagged with mCherry (5.9 × 10^12^ vg/mL) or AAV2/hSyn-DIO-hM4D(Gi) tagged with mCherry (5.60 × 10^12^ vg/mL; both Virus Vector Core, University of North Carolina). The cre-dependent, AAV-DIO-synaptophysin-mCherry (3 × 10 ^12^ vg/mL) (kindly gifted by Dr Martin Myers, University of Michigan, USA) used for anatomical tracing has been described previously (6). Red-fluorescent microspheres (0.04 μm, Cat. No. F8793, Life Technologies, UK) were used to confirm the targets identified via AAV-DIO-synaptophysin-mCherry tracing. *Pacap*-cre::EYFP mice were anesthetized using isoflurane and placed in a stereotaxic frame (Kopf Instruments, Tujunga, CA, USA). The skull was exposed using a small incision followed by craniotomy. Viruses or tracers were delivered (unilaterally for tracing and bilaterally for chemogenetic manipulation using DREADDs) through a pulled glass micropipette controlled by a nanoinjector (Nanoinject II Auto-Nanoliter Injector, Drummond Scientific Company, Pennsylvania, USA) using co-ordinates based on the Mouse Brain Atlas (16); VMH, AP −1.1 mm, ML ±0.5 mm, DV −6.0 mm; aBNST, AP +0.5 mm, ML ±0.6 mm, DV −4.7 mm; PVH, AP −0.9 mm, ML ±0.2 mm, DV −5.4 mm; PVT, AP −0.46 mm, ML±0.05 mm, DV −3.45 mm; LH, AP −1.3 mm, ML ± 1.3 mm, DV −5.2 mm; PAG, AP −3.08 mm, ML ±0.15 mm, DV −3.5 mm. Mice injected with viruses or retrobeads were left in their home cages for 2 weeks to allow for cell transduction and/or axonal transport. They were handled daily for at least 1 week before any *in vivo* manipulations were carried out.

### DREADD experiments

To understand the effect of activating the cell bodies, PACAP^VMH^ neurons were transfected with the stimulatory DREADD, AAV8/hSyn-DIO-hM3D(Gq). Two weeks later, baseline blood glucose was measured followed by a 1 mg/kg clozapine-N-oxide (CNO) (Cat. No. 6329, Tocris Biosciences, Bristol, UK) injection intraperitoneal (i.p.). CNO for injection was freshly prepared on the day of the test in sterile saline. Glucose levels were monitored at 30, 60, 90 and 120 min after CNO using a glucometer (CodeFree, SD Biosenser, Korea). In a separate experiment, serum samples were collected 30 min after saline or CNO injections and quantified using Ultra Sensitive Mouse Insulin ELISA kit (90080, Crystal Chem, IL, USA) and Mercodia Glucagon ELISA kit-10 μl (10-1281-01, Uppsala, Sweden). For the intraperitoneal glucose tolerance test (IPGTT), mice transfected with hM3Dq, were fasted from 9 a.m. to 1 p.m. on the test days. Following the protocol of Steculorum et al. (17), mice were given two injections of CNO separated by 1 h. Immediately after the second injection, baseline glucose was measured and the mice were given 2 g/kg of glucose (i.p.). Further glucose measurements were made at 15, 30, 60, 90, and 120 min after CNO or control saline injections. To demonstrate that CNO injection was not producing an effect by itself, wild-type C57BL/6J mice were handled for 2 weeks before carrying out the same IPGTT protocol. To carry out the insulin tolerance test (ITT), mice transfected with the inhibitory DREADD (hM4Di), were fasted from 9 a.m. until 1 p.m. on the test days. Baseline blood glucose was measured followed by a 1 mg/kg CNO injection (i.p.). 30 min after the first injection, the mice were given subcutaneous injections of 1.75 U/kg Insulin (Humulin, Eli Lilly and Company Ltd). Further glucose measurements were made at 30, 60, 90, and 120 min after insulin injections. Data were analyzed and plotted using GraphPad Prism Version 7.

### Dual-label immunohistochemistry

Mice were anesthetized with isoflurane and perfused transcardially with 0.9% NaCl containing heparin followed by 4% paraformaldehyde in phosphate buffer (PB). The brain was removed into the same fixative overnight followed by 30% sucrose solution for another 3 days. 30 μM coronal brain sections were cut using a freezing sledge microtome and dual labeling was performed as described previously (18). Briefly, free-floating sections were permeabilized in Tris-PB (0.2% Triton X-100 (Sigma Aldrich, UK) in 0.1 M PB) followed by an overnight incubation with primary antibodies: 1:1,000 anti-GFP (ab13970, Abcam, Cambridge, UK) for EYFP, 1:1,000 anti-nitric oxide synthase 1 (AB5380, Merck Millipore, UK), 1:2,500 anti-DsRed (632496, Clontech Laboratories, Inc, CA, USA) for mCherry staining and 1:500 cFos (sc-52, Santa Cruz Biotechnology, Dallas, TX, USA). Primary antibodies were visualized using 1:1,000 Alexa fluor secondary antibodies A11039 and A11037, Invitrogen. For cFos staining, signal was amplified using Biotin-SP-conjugated secondary antibody (711-065-152, Jackson ImmunoResearch Laboratories, Pennsylvania, USA), and Alexa tagged streptavidin (016-540-084, Jackson ImmunoResearch Laboratories, Pennsylvania, USA). Images were taken using an Axio Imager.D2 upright fluorescent microscope (Zeiss, Sweden) and micrographs captured using a Coolsnap HQ2 camera (Photometrics, Tucson, AZ, USA) via Micromanager software v1.4.23 (https://micro-manager.org/). The images were further processed in ImageJ (19) and merged in Corel Paint Shop Pro Photo X2 (Corel, Ottawa, Canada).

### Statistical analysis

All data sets were analyzed and plotted as mean±standard error of mean using GraphPad Prism version 7. Electrophysiological recordings were analyzed using either one-way ANOVA or paired *t*-tests and significance was set at *p*-value < 0.05. IPGTT and ITT data were analyzed using repeated measure two-way ANOVA with Sidak *post-hoc* comparisons. Hormone data changes were analyzed using unpaired *t*-tests.

## Results

**Figure 1:**
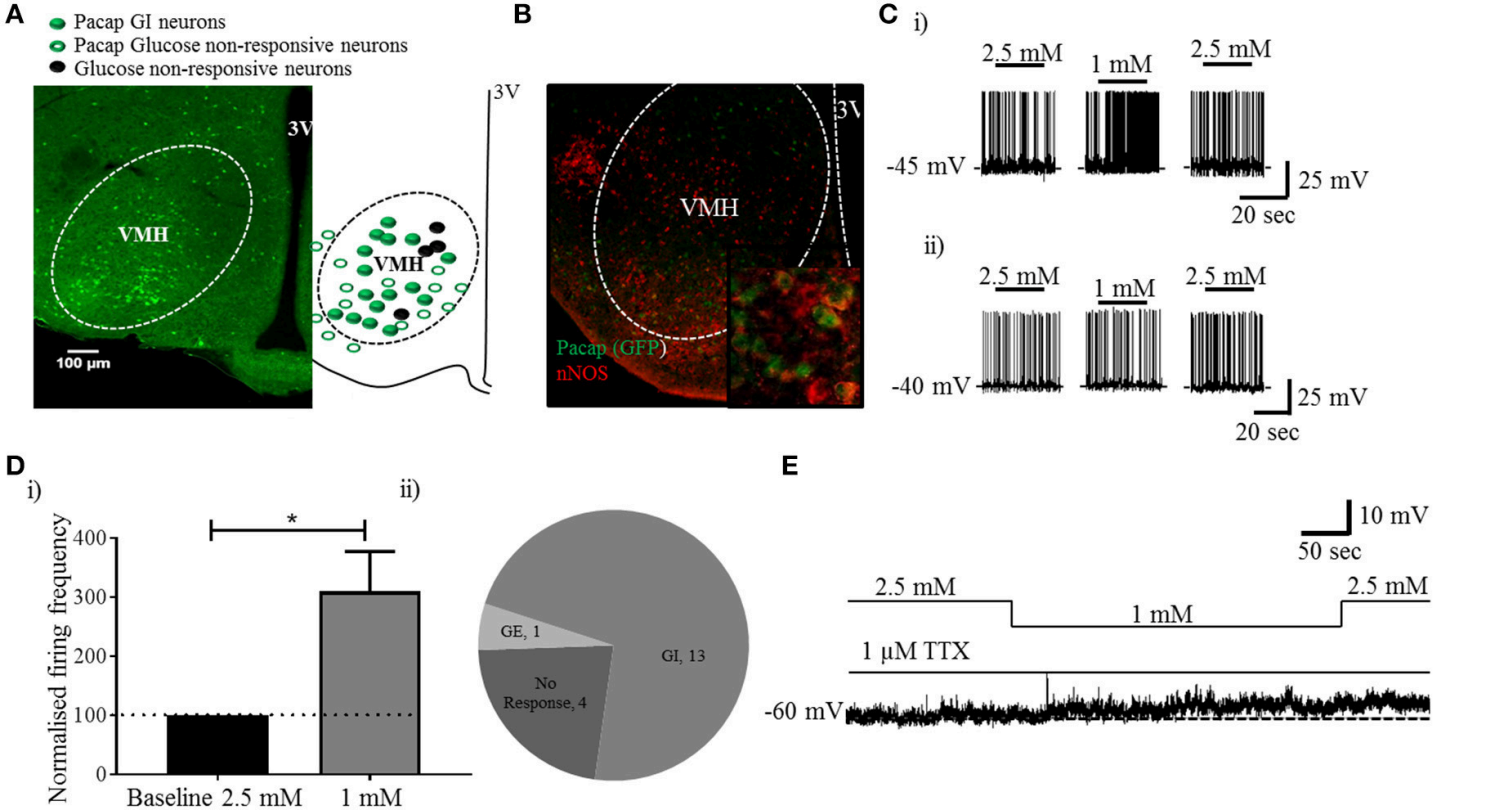
PACAP neurons inside, but not outside the VMH are GI: analysis of PACAP neurons from *Pacap*-cre::EYFP mice. **(A)** Representative coronal brain section from a *Pacap*-cre::EYFP mouse. The accompanying sketch summarizes the distribution of glucose-responsive and non-responsive neurons (PACAP and non-.PACAP) in the ventromedial hypothalamus. **(B)** Representative image (inset higher magnification) of dual-label immunohistochemistry showing co-localization of nNOS labeling with PACAP^VMH^. **(C)** Whole-cell patch-clamp recordings showing the response to a change in glucose (2.5 mM to 1 mM) on PACAP neurons inside (i) and outside (ii) the VMH. **(D)** (i) Quantitative (increase in baseline firing frequency; paired *t*-test, ^*^p < 0.05, *n* = 8) and (ii) qualitative analysis of PACAP^VMH^ neuron response to low glucose. **(E)** The neurons respond to low glucose with depolarization in the presence of TTX.

**Figure 2:**
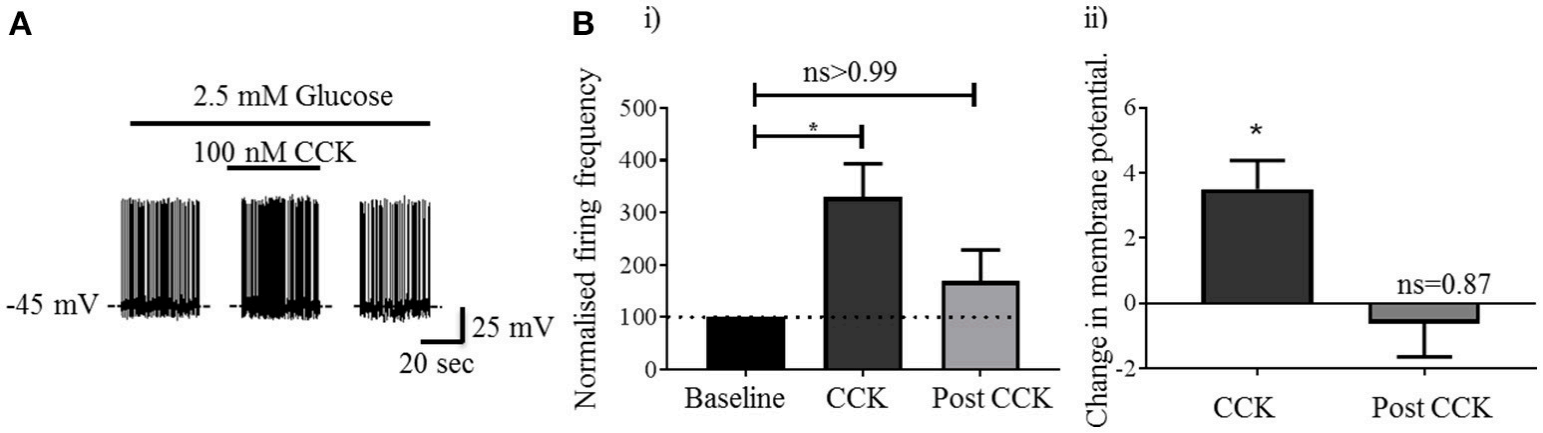
Activation of PACAP^VMH^ neurons by local administration of CCK: **(A)** Whole-cell patch-clamp recording of a PACAP^VMH^ neuron following CCK application (100 nM in 2.5 mM glucose). **B** (i) CCK application transiently increased firing frequency compared with baseline (^*^p < 0.05, one-way ANOVA with repeated measures, *n* = 9 cells from 7 mice). **B** (ii) A concomitant increase in membrane potential following CCK application (^*^p < 0.05, one-way ANOVA with repeated measures, *n* = 8 from 7 mice).

Visualization of sections from *Pacap*-cre::EYFP mice confirmed that PACAP-containing neurons have a widespread distribution in the forebrain (14) including in many nuclei of the hypothalamus (Figure 1A). Using dual-label immunohistochemistry, we observed that PACAP-EYFP is expressed in 15% of neurons staining for neuronal nitric oxide synthase 1 (nNOS) within the VMH (Figure 1B). Whole-cell patch-clamp electrophysiology demonstrated that 13/18 PACAP^VMH^ neurons responded to a decrease in glucose (2.5 mM to 1 mM) with an increase in firing rate (average change from 2.5 mM 0.70±0.2 Hz to 1 mM 1.3±0.2 Hz), which was reversed when 2.5 mM glucose was reinstated (Figures 1C,D). Four PACAP^VMH^ neurons did not respond, and one showed a slight decrease in firing rate. By contrast, 2/10 PACAP neurons outside the VMH, in the area immediately surrounding the nucleus, responded to glucose. PACAP^VMH^ neurons were tested in the presence of TTX to block action potentials. 5/6 neurons still responded to low glucose with depolarization (Figure 1E and Figure S1), demonstrating that they are intrinsically GI. Also, since the VMH receives input from other glucose-sensing regions of the brain, including from cholecystokinin (CCK)-containing neurons in the brainstem parabrachial nucleus (5, 6), we wished to test if PACAP^VMH^ neurons could integrate such information 19/21 PACAP^VMH^ neurons responded to CCK with a transient depolarization of their membrane potential and an increase in firing rate (Figure 2).

**Figure 3:**
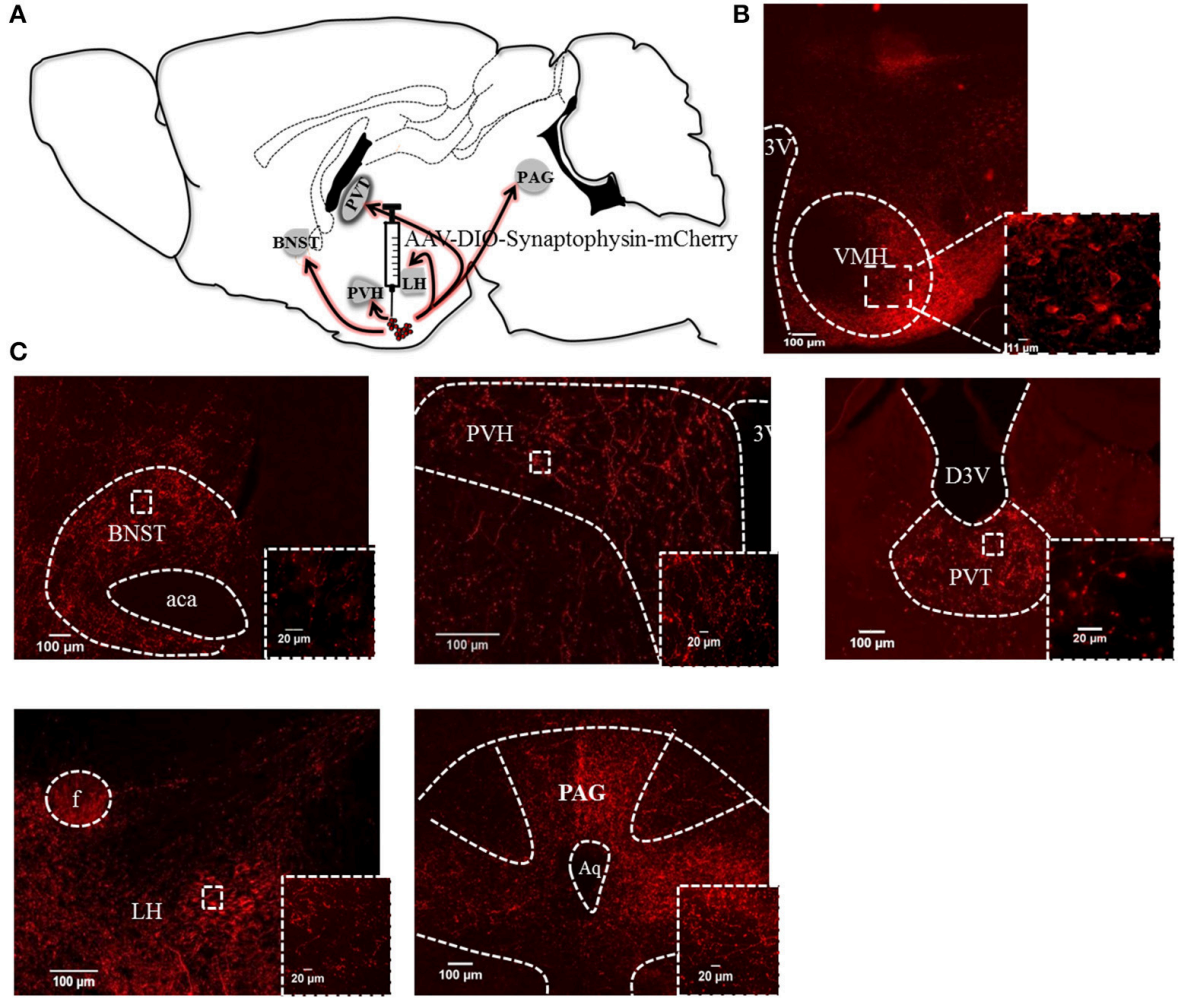
Efferent anatomical projections of PACAP^VMH^ neurons: **(A)** Summary of efferent connections in *Pacap*-cre::EYFP mice using expression of AAV-driven synaptophysin tagged with mCherry. **(B)** Fluorescent micrograph showing PACAP^VMN^ transfected with synaptophysin virus. **(C)** Fiber staining showing projections to the anterior BNST (aBNST), paraventricular hypothalamic nucleus (PVH), paraventricular thalamic area (PVT), lateral hypothalamus (LH) and periaqueductal gray (PAG) were observed. Inset higher magnification show axonal terminals to the above mentioned area.

Next we injected *Pacap*-cre mice with a cre-dependent, AAV-DIO-synaptophysin-mCherry virus (6) unilaterally into the VMH. Synaptophysin is a synaptic vesicle protein and, thus, the mCherry-tagged transgenic protein is transported to the terminals of PACAP^VMH^ neurons and can be used as an anterograde neuroanatomical tracer. Sections from the whole brain were cut and analyzed for synaptophysin-mCherry staining. Cell bodies were labeled in the injected VMH, as were synaptic boutons, suggesting that PACAP neurons terminate within the nucleus itself. There was sparse staining in many parts of the hypothalamus, but very strong staining was observed in the paraventricular nucleus (PVH) and the lateral hypothalamic (LH) area (Figure 3). The PVH, in particular, had dense boutons ipsilateral to the injection site though terminals were clearly visible on both the ipsilateral and contralateral sides. Three other regions of the brain demonstrated strong terminal staining: the anterior part of the bed nucleus of the stria terminalis (aBNST), the paraventricular nucleus of the thalamus (PVT) and throughout the periaqueductal gray (PAG). To confirm direct projections, each of the five main target regions were injected with red-fluorescent, latex microbeads unilaterally in separate *Pacap*-cre::EYFP mice. These microbeads are taken up by nerve terminals in the vicinity of the injection site, and transported retrogradely to the cell bodies of origin. In this way, we confirmed that PACAP^VMH^ neurons project directly to the PVH, LH, aBNST, PVT, and ventrolateral PAG (Figure S2).

**Figure 4:**
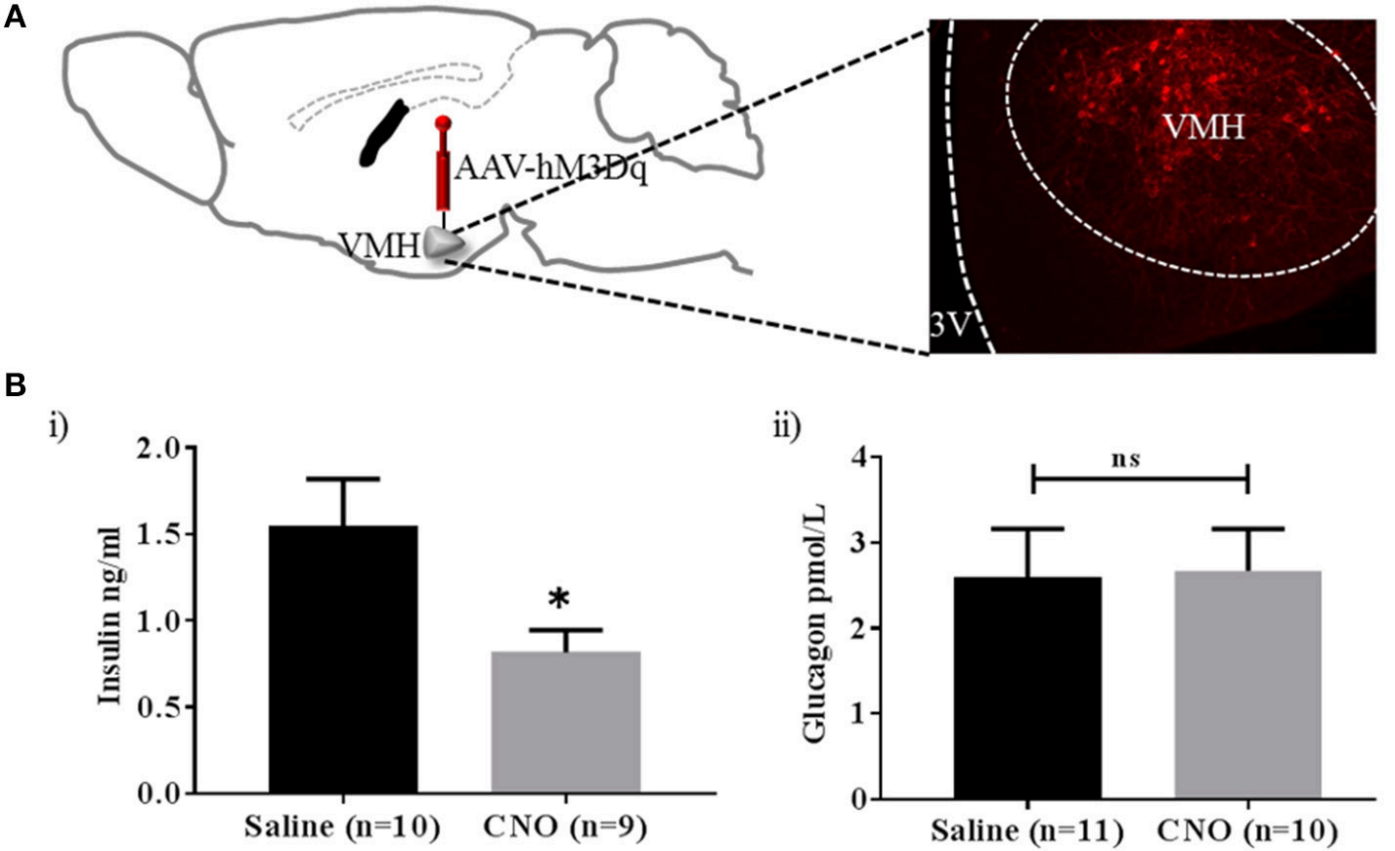
Activation of PACAP^VMH^ neurons causes inhibition of insulin release: **(A)** Schematic sketch representing intracranial, bilateral injection of cre-dependent stimulatory DREADDs in *Pacap*-cre mice. **(B)** Change in glucose-sensitive hormones following CNO injections: (i) decrease in insulin (ii) no change in glucagon response. Data are analyzed using unpaired *t*-test (^*^p < 0.05).

From the results so far, PACAP^VMH^ neurons are a relatively small population of cells in the VMH, which project to regions of the brain that may affect glucose homeostasis (see section Discussion). Currently, we do not know if individual neurons project to one or several of these target areas. Therefore, we aimed to activate as many PACAP^VMH^ neuronal cell bodies *in vivo* as possible using a chemogenetic approach. GI cells have been implicated in the counter-regulatory response to hypoglycaemia and, therefore, their activation is predicted to increase circulating glucose. In an initial experiment, the cre-dependent DREADD, AAV8/hSyn-DIO-hM3D(Gq), was injected bilaterally in the VMH of *Pacap*-cre mice. Only animals with bilateral transduction of PACAP^VMH^ neurons (Figure S3) were included in analysis, and CNO caused a delayed increase in baseline glucose levels (Figure S4). To confirm neuronal activation, later half the mice were given a further injection of CNO and perfused to allow immunohistochemistry for cFos. This demonstrated a clear activation of PACAP^VMH^ neurons (Figure S5). We then repeated this experiment, but this time measured circulating hormone levels. Importantly, a decrease in plasma insulin compared with control mice was observed following CNO injection, whereas glucagon levels were not altered (Figure 4).

**Figure 5:**
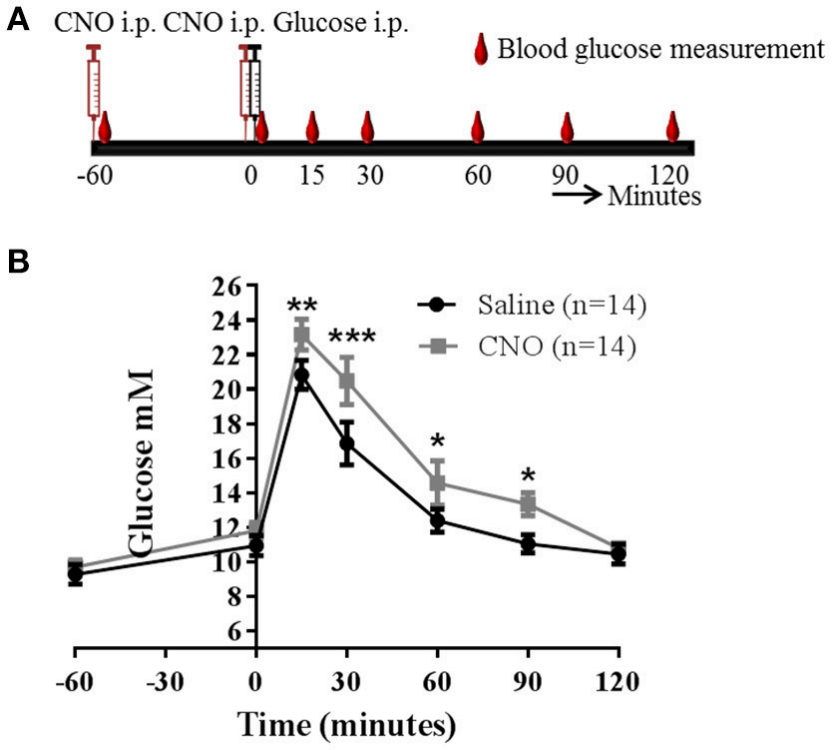
Activation of PACAP^VMH^ neurons causes an increase in glucose during a glucose tolerance test (IPGTT): *Pacap*-cre mice were given intracranial, bilateral injection of cre-dependent stimulatory DREADDs. **(A)** Schematic sketch representing the protocol used for an IPGTT experiment. **(B)** Two weeks post-surgery, mice were injected with either saline or CNO followed by an IPGTT. Data are analyzed using two-way ANOVA with repeated measures (^*^p < 0.05, ^**^p < 0.01 and ^***^p < 0.001).

This suggested to us that the role of PACAP neurons in glucose regulation may be via a hormonal response, likely by inhibiting insulin release. To confirm this possibility, we chose the IPGTT model, since it is a situation when the mice secrete insulin in response to glucose administration. Also, in this scenario GI neurons would normally be inhibited and hypoglycaemia counter-regulatory hormones low. Control mice given the IPGTT displayed the expected increase in circulating glucose which peaked around 15 min and returned to baseline by 60 min. Mice pre-injected with CNO demonstrated impaired glucose tolerance, as predicted: there was a greater plasma glucose excursion (Figure 5). However, since concerns have been raised recently that CNO can, in certain circumstances, be broken down to the active drug clozapine and have off-target effects of its own (20–22), we carried out a control experiment in which wild-type C57BL/6J mice were treated in the same way. In this case, treating the mice with CNO did not affect the IPGTT (Figure S6).

Lastly, we wished to confirm that PACAP^VMH^ neurons are not affecting glucagon secretion, for which an IPGTT cannot be used since the hyperglycaemia itself would reduce this hormone. Therefore, instead, we carried out an insulin tolerance test (ITT) while inhibiting PACAP^VMH^ neurons with AAV2/hSyn-DIO-hM4D(Gi). Further supporting our earlier results, no differences in circulating glucose or glucagon levels following DREADD-induced inactivation were observed in a situation of high circulating insulin (Figure S7).

## Discussion

A full understanding of how the brain maintains homeostasis can only be gleaned once individual neurons underpinning a mechanism have been identified. Our knowledge of the central regulation of glucose has lagged behind that of other homeostatic systems, such as those which control appetite, sleep or plasma electrolytes, despite the fact that the main location of specialist, glucose-sensing neurons has been known for over 50 years (23). The VMH is a complex structure with many functions, including in feeding, sexual, and aggressive behavior (7, 24–26), but it also has the highest density of neurons which can respond to small changes in ambient glucose. In fact, the VMH experiences glucose levels much lower than those circulating in the bloodstream [the physiological range experienced in the healthy rodent probably lies between 0.15 and 2.5 mM (27, 28)]. This realization has led to the redefinition of true, inherent glucose sensing, but with the VMH still containing the highest density of intrinsically sensitive GE and GI cells. Despite this, the identity of any single population has remained elusive. Meanwhile, direct evidence has been accumulating for the existence of intrinsically sensitive neurons elsewhere in the brain, including GE cells (for example, melanin-concentrating hormone neurons in the LH) (29) and GI cells (for example, orexin/hypocretin neurons in the LH) (29), glucose transporter 2 neurons in the PVT (30) and leptin-receptive CCK neurons in the parabrachial nucleus (6, 31).

Attempts have been made to define populations of VMH neuron by their expression of proteins hypothesized to distinguish the ability to sense glucose. For example, the low-affinity glucokinase and the K_ATP_ channel, which are key to the glucose sensitivity of pancreatic β cells, have been implicated in glucose sensing by neurons (32, 33). Indeed, the electromagnetic or optogenetic activation of glucokinase-expressing cells in an extended region of the ventromedial hypothalamus (including the arcuate, ventromedial, and dorsomedial nuclei) will increase circulating glucose (7). However, at best, these markers are found in mixed populations of GE, GI, and non-glucose sensing cells (32). Perhaps the best distinguishing marker in the VMH is nNOS, which appears to be a molecular requirement for GI, but not GE cells in this nucleus (11, 34), though it is not clear if nNOS is also found in non-sensing neurons within the VMH, as it clearly is outside the nucleus. Here we have identified what appears to be a single, identifiable population of glucose-sensing VMH cell. Dual-labeling indicates that PACAP neurons in the VMH are one type of nNOS-positive GI cell. Recent publications have postulated that GI CCK neurons in the brainstem parabrachial nucleus send a monosynaptic connection to the VMH which can engage counter-regulatory mechanisms in response to hypoglycaemia (5, 6, 31). And, since all CCK input to the VMH is believed to come from the parabrachial nucleus (35), it is relevant that PACAP^VMH^ neurons increase their activity in response to CCK and, thus, are responsive both directly and indirectly to hypoglycaemic stimuli.

VMH neurons are most sensitive to changes in glucose at the lower end of the range and the responses are activated following rapid, rather than to slow-onset hypoglycaemia (36). Thus, it is likely that PACAP^VMH^ neurons are involved in protecting the brain against glucoprivation rather than the maintenance of normal, inter-meal glucose levels. Here, we have demonstrated that PACAP^VMH^ neurons project densely within the VMH, and externally to the PVH, LH, aBNST, PVT, and PAG, which compares with the general projection profile noted for the mixed population of SF-1-positive neurons by Morton et al. (5). They describe SF-1 projections to the PVH, aBNST, central amygdala (CeA) and PAG. We might suggest that PACAP^VMH^ neurons can project to downstream effector regions directly, but also indirectly, perhaps via a major population of SF-1-positive PED neurons in the dorsomedial VMH. This is comparable to recently described PACAP neurons in the central VMH which synapse within the ventrolateral VMH, possibly connecting circadian rhythmicity onto aggressive behaviors (26).

Although the optogenetic stimulation of all SF-1 neurons together can increase circulating glucose (5), this is a compound response since, by definition, SF-1 is expressed in multiple populations. These populations can normally respond in opposite directions to glucose, or have no role in normal glucoregulation, but can produce additional unrecorded behaviors, such as avoidance or aggression (25, 26) which may or may not affect glucose levels indirectly. We now show that chemogenetic stimulation of PACAP^VMH^ cell bodies alone is sufficient to increase circulating glucose in the setting of a glucose-tolerance test, probably via inhibition of insulin release. Morton et al. (5) found that optical stimulation of all SF-1 cell bodies increased glucose in this situation, apparently by both inhibiting glucose-induced insulin release and by causing the secretion of the counter-regulatory hormones, glucagon and corticosterone. Here, we found no effect on circulating glucagon. It is possible that the compound glucose response to SF-1 cell stimulation is dominated by the output of PED neurons located mainly in the dorsomedial VMH, which may be the most responsive to optogenetic stimulation, or which may produce the strongest downstream response. The issue of activating this mixed population is further complicated by an independent study which found that chemogenetic activation of all SF-1 neurons actually decreased glucose during a glucose tolerance test, by increasing glucose uptake into skeletal muscle (37).These discrepancies highlight the need to identify and manipulate single-cell populations in the VMH.

Morton et al. (5) went on to show that optical stimulation of SF-1 terminals in the aBNST, but not in the PVH, CeA, or PAG, induced an increase in circulating glucose concomitant with the secretion of glucagon and corticosterone, and the inhibition of insulin. We have demonstrated significant projections from PACAP^VMH^ neurons to the aBNST and PVH, but also to the LH and PVT. Since PACAP^VMH^ neurons are intrinsically GI they are almost certain to be a key element in glucose regulation. However, because they are a relatively sparse population and likely to influence effector sites both directly and indirectly (through local VMH connections), it may make them difficult to interrogate specifically using optogenetic stimulation of terminal fields. Morton et al. (5) did report that optical stimulation of SF-1 terminals in the PVH caused a non-significant increase in corticosterone. Thus, the PACAP^VMH^ → PVH pathway is worth further investigation, not least because Kalsbeek and colleagues have previously shown that PACAP, of unknown source, can act in the PVH to increase plasma glucose levels and endogenous glucose production (13). Likewise, direct PACAP^VMH^ projections to the LH are also interesting. The LH is the location of GI orexin/hypocretin neurons which are also positioned to affect many aspects of hypoglycaemia counter regulation, including a potential role in a behavioral response leading to increased feeding (38–40). Also, glucose transporter 2-containing neurons in the PVT are glucose sensing and can initiate sucrose seeking (30). Our identification of the first phenotypically-defined, glucose-sensing neuron in the VMH, the key nucleus involved in the responses to hypoglycaemia, will help us to piece together the neuronal network involved in glucose homeostasis.

## Author contributions

TK designed, carried out the experiments, analyzed, interpreted the data, and wrote the manuscript with input from all the authors. NN designed, carried out the experiments, contributed to interpretation of the data. MB designed, contributed to interpretation of the electrophysiology data. AW and CF contributed to the experiments and helped with interpretation of the data. HP edited the manuscript. SL supervised, designed, interpreted, wrote and edited the manuscript.

### Conflict of interest statement

The authors declare that the research was conducted in the absence of any commercial or financial relationships that could be construed as a potential conflict of interest.

## Supplementary material

Figure S1 Changes in membrane potential following TTX application: a significant increase in membrane potential in response to low glucose in the presence of TTX was observed. Data are analyzed using paired *t*-test (^*^*p* < 0.05), *n* = 6.

Figure S2 Confirmation of observed efferent PACAP^VMH^ connections**:** efferent connections of PACAP^VMH^ neurons to the aBNST, PVH, LH, and PAG, previously observed using AAV-driven synaptophysin, were confirmed here using retrograde tracing. For each target region, the injection site is shown in the left panel and retrobead-containing PACAP^VMH^ neurons in the right panel.

Figure S3 Bilateral transfection in VMH: only mice showing bilateral transfection with the stimulatory DREADD, AAV8/hSyn-DIO-hM3D(Gq), were included in the analysis.

Figure S4 Changes in baseline glucose levels following activation of PACAP^VMH^ neurons: 8–10 week old, male mice were injected with saline or CNO (i.p.) and changes in baseline glucose levels measured. A transient increase in baseline glucose levels following CNO injections was observed in mice after CNO injection.

Figure S5 cFos in PACAP^VMH^ neurons using DREADD**:** cFos expression (green) in PACAP^VMH^ neurons following **(A) (i)** Saline or **(ii)** CNO injections. **(B)** A significant increase in mCherry tagged neurons containing cFos was observed following CNO injections, compared with controls. Data are analyzed using unpaired *t*-test, ^****^*p* < 0.0001.

Figure S6 Effect of CNO in C57BL/6J: 8–10 weeks, male mice were injected with either saline or CNO followed by an IPGTT. No difference in **(A) (i)** glucose profiles and **(ii)** AUC was observed between the two groups. Two-way ANOVA with repeated measure and unpaired *t*- test, *n* = 6 in each group.

Figure S7 Inhibition of PACAP^VMH^ neurons causes no change in glucose or glucagon during an insulin tolerance test (ITT): **(A)** Silencing of PACAP^VMH^ neurons by CNO pretreatment did not change insulin-induced glucose response compared with controls. Data are analyzed using two-way ANOVA with repeated measure (^****^*p* < 0.0001). **(B)** Glucagon levels were unchanged after CNO injections compared with controls. Data are analyzed using unpaired *t*-test.
