# Supplementary figures and images for: PACAP Neurons in the Ventromedial Hypothalamic Nucleus Are Glucose Inhibited and Their Selective Activation Induces Hyperglycaemia
Source: Front Endocrinol (Lausanne). 2018 Oct 30;9:632. doi: 10.3389/fendo.2018.00632 (PMC6218416; doi:10.3389/fendo.2018.00632)

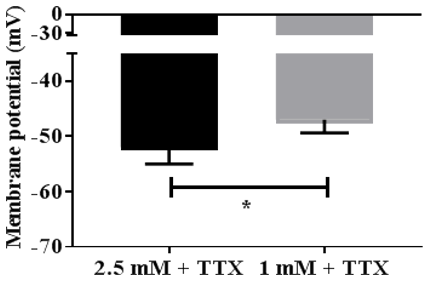

Supplement: Figure S1 — Changes in membrane potential following TTX application: a significant increase in membrane potential in response to low glucose in the presence of TTX was observed. Data are analyzed using paired t-test (*p < 0.05), n = 6. [file Image_1.TIF]

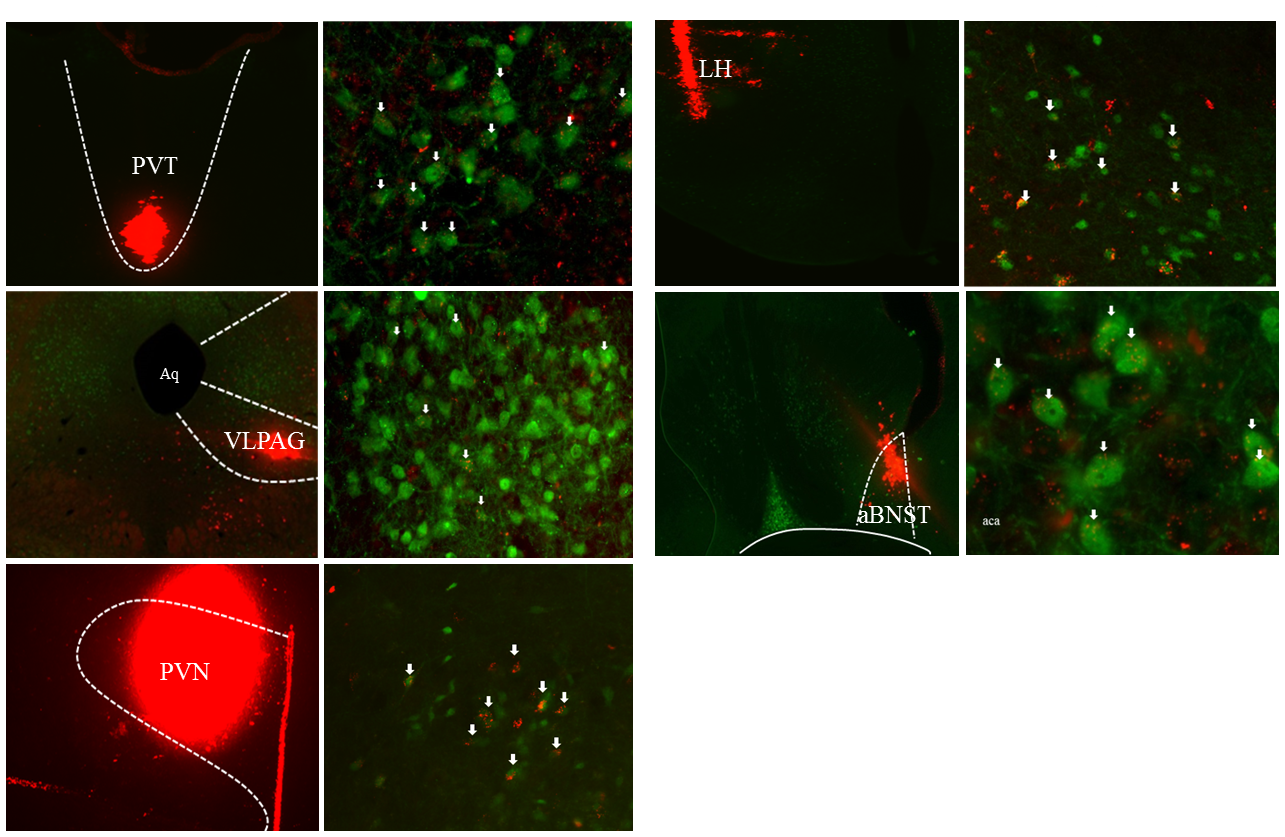

Supplement: Figure S2 — Confirmation of observed efferent PACAPVMH connections: efferent connections of PACAPVMH neurons to the aBNST, PVH, LH, and PAG, previously observed using AAV-driven synaptophysin, were confirmed here using retrograde tracing. For each target region, the injection site is shown in the left panel and retrobead-containing PACAPVMH neurons in the right panel. [file Image_2.TIF]

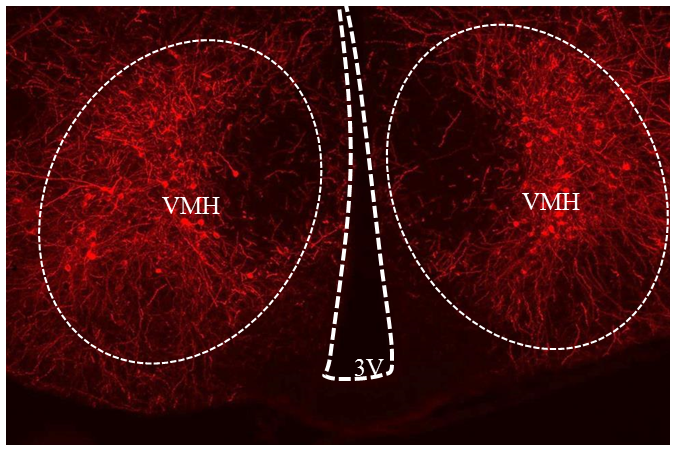

Supplement: Figure S3 — Bilateral transfection in VMH: only mice showing bilateral transfection with the stimulatory DREADD, AAV8/hSyn-DIO-hM3D(Gq), were included in the analysis. [file Image_3.TIF]

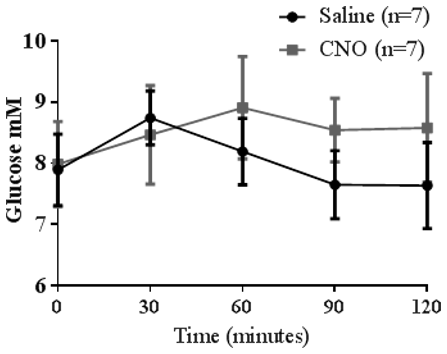

Supplement: Figure S4 — Changes in baseline glucose levels following activation of PACAPVMH neurons: 8–10 week old, male mice were injected with saline or CNO (i.p.) and changes in baseline glucose levels measured. A transient increase in baseline glucose levels following CNO injections was observed in mice after CNO injection. [file Image_4.TIF]

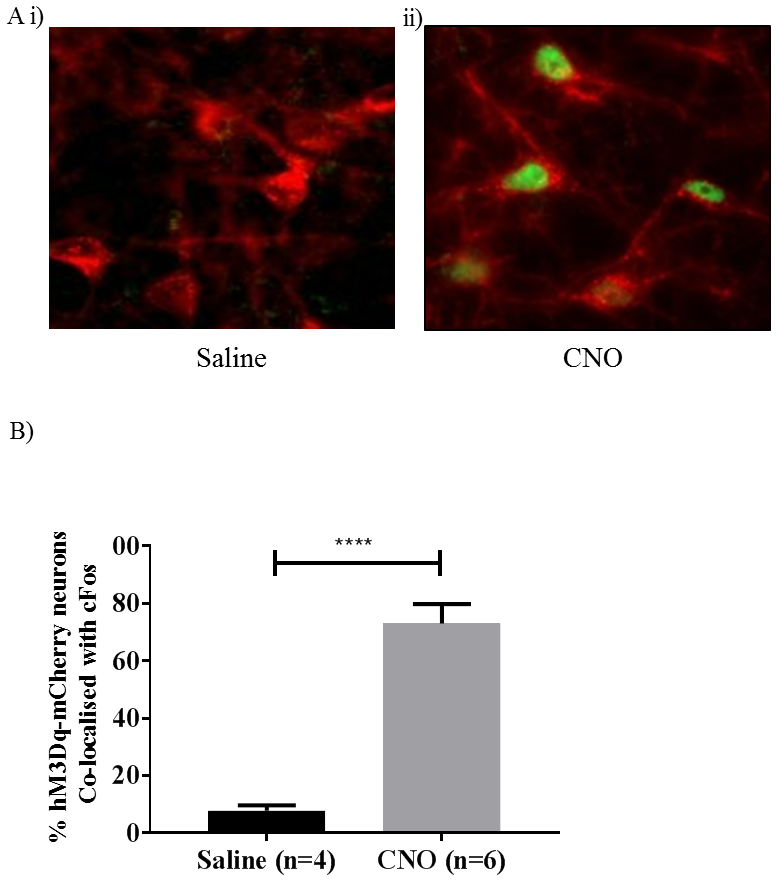

Supplement: Figure S5 — cFos in PACAPVMH neurons using DREADD: cFos expression (green) in PACAPVMH neurons following (A) (i) Saline or (ii) CNO injections. (B) A significant increase in mCherry tagged neurons containing cFos was observed following CNO injections, compared with controls. Data are analyzed using unpaired t-test, ****p < 0.0001. [file Image_5.TIF]

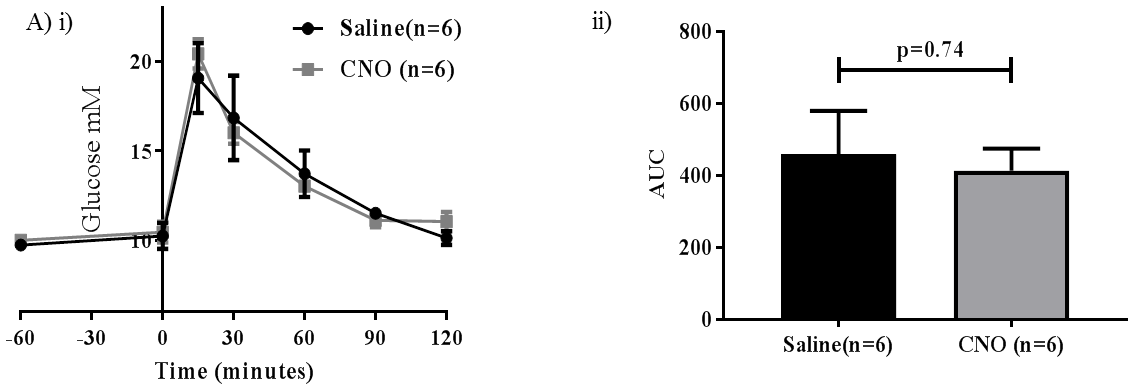

Supplement: Figure S6 — Effect of CNO in C57BL/6J: 8–10 weeks, male mice were injected with either saline or CNO followed by an IPGTT. No difference in (A) (i) glucose profiles and (ii) AUC was observed between the two groups. Two-way ANOVA with repeated measure and unpaired t- test, n = 6 in each group. [file Image_6.TIF]

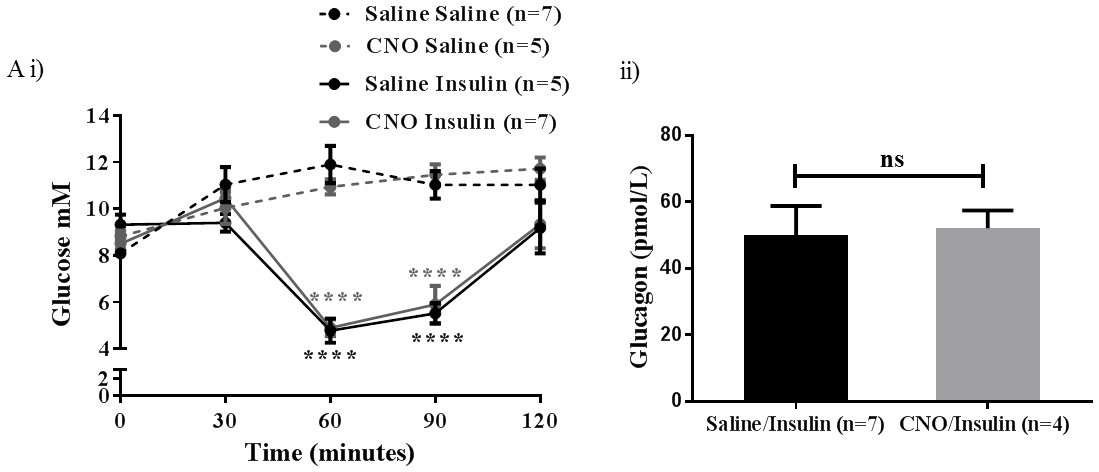

Supplement: Figure S7 — Inhibition of PACAPVMH neurons causes no change in glucose or glucagon during an insulin tolerance test (ITT): (A) Silencing of PACAPVMH neurons by CNO pretreatment did not change insulin-induced glucose response compared with controls. Data are analyzed using two-way ANOVA with repeated measure (****p < 0.0001). (B) Glucagon levels were unchanged after CNO injections compared with controls. Data are analyzed using unpaired t-test. [file Image_7.TIF]
